# A Rare Case of Isolated Psoas Muscle Echinococcal Cyst

**DOI:** 10.7759/cureus.88577

**Published:** 2025-07-23

**Authors:** Yuan Lu, Liuyang Zhan, Huwei Jiang, Xiaofeng Li, Chao Jiang

**Affiliations:** 1 General Surgery, Affiliated Hospital of Qinghai University, Xining City, CHN; 2 Urology, Affiliated Hospital of Qinghai University, Xining City, CHN

**Keywords:** echinococcosis, intramuscular hydatid cyst, isolated, parasitic infection, surgical treatment of hydatid cyst

## Abstract

Echinococcosis is a rare zoonotic parasitic disease characterized by space-occupying lesions in affected organs. Most of the cases involve the liver, while muscular invasion is relatively uncommon. The disease is typically asymptomatic in early stages and often detected only when cyst enlargement causes compression of adjacent organs or rupture. We present a case of a 36-year-old male pastoralist (with cattle/sheep exposure) with intermittent right flank pain as the presenting symptom. Contrast-enhanced computed tomography (CECT) revealed two thin-walled cystic lesions within the right iliopsoas muscle. Serological testing by enzyme-linked immunosorbent assay (ELISA) confirmed specific IgG antibodies against* Echinococcus granulosus*. The patient subsequently underwent open cystectomy with uneventful recovery. Histopathological examination confirmed echinococcosis. For patients presenting with chronic low back pain in endemic areas, atypical infections like echinococcosis must remain a part of the differential diagnosis despite their rarity.

## Introduction

Hydatid cyst, scientifically referred to as echinococcosis cyst, is a significant zoonotic parasitic infection caused by the larval stage of cestodes belonging to the genus *Echinococcus*, primarily cystic echinococcosis and alveolar echinococcosis. Hydatid cysts are endemic in 149 countries worldwide, with primary distribution in pastoral regions including Western China, Central Asia, South America (Argentina, Brazil), Mediterranean countries, and East Africa. The liver and lungs are overwhelmingly the primary sites of involvement, harbouring approximately 80-85% and 10-15% of infection foci [1]. In stark contrast, musculoskeletal involvement by *Echinococcus *larvae represents an exceptionally rare manifestation of the disease. Relevant research data indicate that the incidence rate of echinococcosis in skeletal muscle is only 2-3% [2].

Consequently, isolated lesions confined exclusively to the deep-seated iliopsoas muscle complex (comprising the psoas major, iliacus, and psoas minor muscles) are extraordinarily uncommon clinical entities, constituting only a fraction of the already rare muscular cases ( <50 cases worldwide). The deep retroperitoneal location of the iliopsoas complex presents inherent diagnostic challenges. This lack of pathognomonic features, combined with the inherent rarity of the condition, leads to significant diagnostic delays. Due to these nonspecific symptoms, the average diagnostic delay reaches 17.3 months [3]. Hydatid disease localized to the psoas muscle region is exceptionally rare and poses significant diagnostic and therapeutic challenges. In this report, we present the complete diagnostic and therapeutic course of a case of psoas hydatid cyst manifesting as lumbar discomfort.

## Case presentation

A 35-year-old male herder from Delingha City, China, reported a two-year history of insidious right flank discomfort. The initial symptoms consisted of intermittent, dull pain localized to the right lower back, exacerbated by weight-bearing activities. He sought evaluation at a local community hospital where oral non-steroidal anti-inflammatory drugs (NSAIDs) provided transient relief, followed by progressive symptom exacerbation. By the 18th month, the patient developed nocturnal pain radiating to the right groin, which proved refractory to oral NSAID therapy.

Physical examination revealed tenderness and percussion tenderness over the right lumbodorsal region and groin. No palpable masses were detected. The patient denied systemic symptoms, including fever, chills, night sweats, headache, vomiting, dysuria, rash, or arthralgia at the time of examination. The patient has a history of long-term residence in a pastoral area with extended exposure to cattle, sheep, and other livestock. There is no history of prior surgery, and all vital signs are within normal limits.

Laboratory investigations revealed elevated levels of direct bilirubin and creatine kinase (CK) in the patient. Given the patient's origin from an endemic region, we performed serological testing for *Echinococcus*-specific IgG antibodies, which yielded positive results. All other laboratory parameters remained within normal limits (Table 1). 

**Table 1:** Laboratory test results. CK: creatine kinase; AST: aspartate aminotransferase; ALT: alanine aminotransferase; ALP: alkaline phosphatase; WBC: white blood cell count

Parameter	Patient's value (reference range）	Result
CK	658 U/L (50.0-310.0 U/L）	High
AST	25 U/L (15.0-40.0 U/L)	Normal
ALT	23 U/L (9.0-50.0 U/L)	Normal
ALP	78 U/L (45.0-125 U/L)	Normal
Total bilirubin	11.8 umol/L (<23.0 umol/L）	Normal
Direct bilirubin	3.7 umol/L (<3.4 umol/L）	High
Serum albumin	42.2 g/L (40.0-55.0 g/L)	Normal
Hemoglobin	16.7 g/dL (13.0-17.5 g/dL)	Normal
Platelet count	286 10⁹/L (125-350 10⁹/L）	Normal
WBC	7.42 10⁹/L (3.5-9.5 10⁹/L)	Normal
Neutrophils (%)	55.2% (40.0-75.0%)	Normal
Lymphocytes (%)	35.2% (20.0-50.0%)	Normal
Eosinophils (%)	4.2% (0.4-8.0%)	Normal

The outpatient abdominal ultrasound examination revealed two cystic masses (measuring approximately 2.5 × 2.0 cm and 3.1 × 3.0 cm) located deep within the right iliopsoas muscle. Given the positive serological results for *Echinococcus*-specific IgG, we established a preliminary diagnosis of hydatid cysts. To better characterize the nature of the mass (benign vs. malignant), we subsequently performed contrast-enhanced computed tomography (CECT) for comprehensive evaluation.

**Figure 1:**
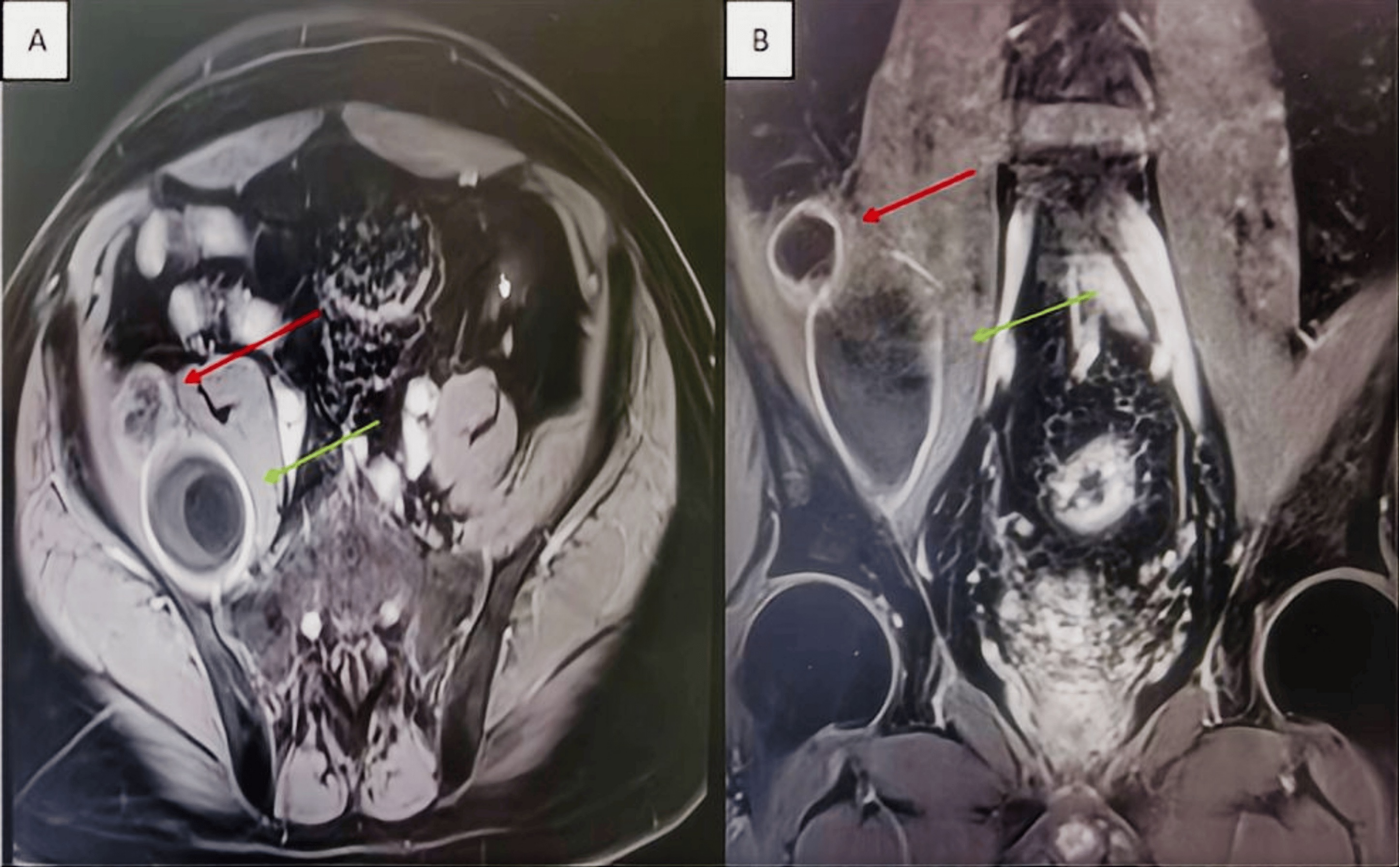
CECT image (A) Axial CECT of the pelvis: superior lesion (denoted by red arrow), inferior lesion (indicated by green arrow). (B) Coronal CECT of the pelvis: superior lesion (red arrow), inferior lesion (green arrow).

The CECT scan displayed two thin-walled cystic lesions within the right iliopsoas muscle. The superior lesion measured approximately 2.6×1.6 cm, and the inferior lesion measured approximately 3.6×3.0 cm. Both exhibited fluid attenuation with well-defined margins and abutted the adjacent lumbar musculature. Post-contrast images showed mild peripheral enhancement of the cyst walls without internal enhancement (Figure 1A, 1B). CECT revealed a cystic mass without typical malignant features (e.g., rapid wash-in/wash-out enhancement) or adjacent bony destruction. Based on the patient's serological and hematological profiles, we preliminarily ruled out malignant neoplasms, tuberculosis, and infected abscesses. To further delineate the anatomical relationship between the lesion and surrounding structures, we performed a comprehensive MRI evaluation.

**Figure 2:**
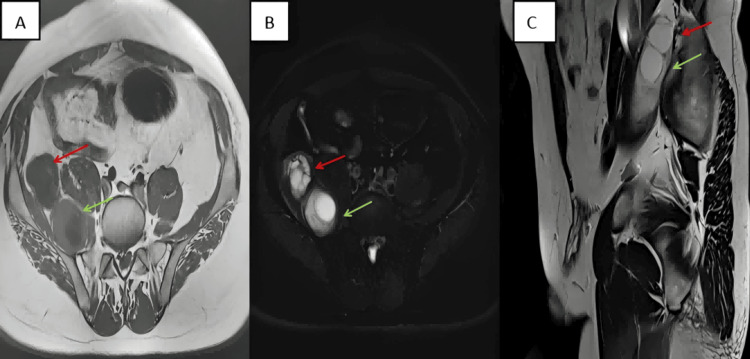
MRI image (A) Axial T1W1 pelvis: superior lesion demonstrates heterogeneous hypointense signal with characteristic cystic signal distribution along the margins(indicated by the red arrow); the inferior lesion shows similar imaging characteristics to the superior lesion(indicated by green arrow). (B) Axial T2W1 pelvis: superior lesion exhibits heterogeneous hyperintense signal with daughter cysts arranged peripherally along the mother cyst wall (red arrow); inferior lesion presents with comparable heterogeneous hyperintense signal (green arrow). (C) Sagittal T2W1 pelvis: superior lesion (red arrow), Inferior lesion (green arrow).

The MRI revealed enlargement of the right iliopsoas muscle, within which multiple irregular lesions of varying sizes were identified. The larger lesions measured 3.5 × 3.2 cm and 3.7 × 3.6 cm, respectively. Daughter cysts are distributed peripherally within the endocyst, forming a multilocular cystic architecture. Diffusion-weighted imaging (DWI) demonstrated mild diffusion restriction within the lesions (Figure 2A-2C).

**Figure 3:**
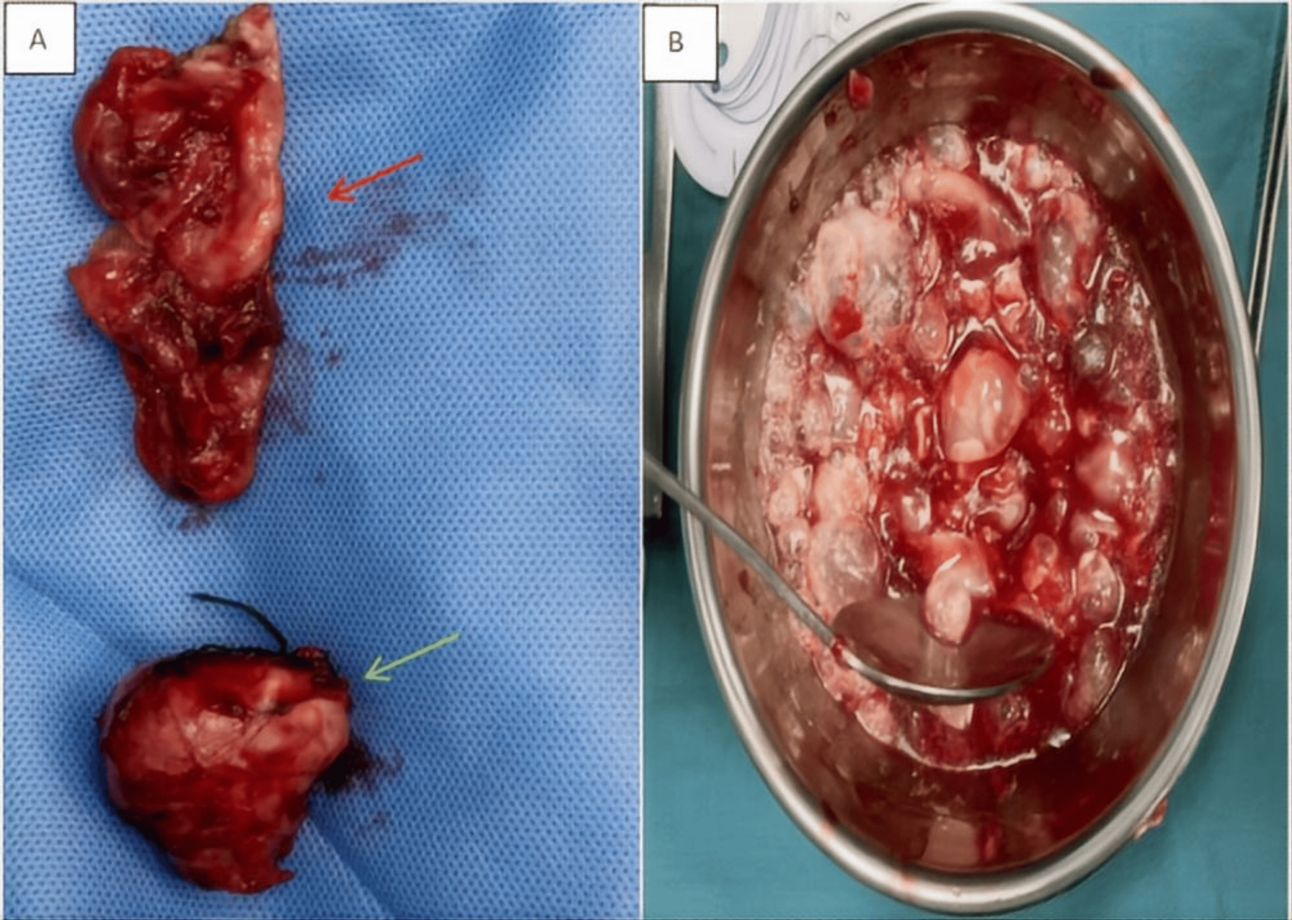
Specimen image Figure A shows a damaged echinococcosis cyst wall (indicated by the red arrow) and a completely exfoliated echinococcosis cyst (indicated by the green arrow). Figure B shows the contents of the damaged echinococcosis cyst.

The diagnosis of multivesicular active cyst​ (CE2) was made based on the presence of positive *Echinococcus*-specific IgG antibodies and characteristic cystic imaging findings. The patient was initiated on ​​preoperative albendazole therapy (400 mg twice daily for one week). Following completion of the pharmacological regimen, the patient underwent ​​exploratory laparotomy via a right pararectal incision under general anesthesia. Intraoperative exploration revealed a cystic mass within the deep iliopsoas compartment. Meticulous examination of the remaining pelvic structures demonstrated no additional pathological lesions. Complete cystectomy was undertaken, with successful en bloc resection of the superior cyst component (Figure 3A). However, the inferior cyst ruptured during dissection due to dense adhesions to the right iliac fossa (Figure 3A-3B). Upon recognizing the cyst rupture, we promptly initiated emergency management: low-pressure suction was employed to aspirate the spilled cystic fluid while hypertonic saline-soaked gauze was used to pack the ruptured site and surrounding tissues. Concurrently, the anesthesiologist was notified to administer 5 mg intravenous dexamethasone and prepare emergency medications for potential anaphylactic shock. All contaminated instruments and drapes were replaced, followed by meticulous en bloc resection of the cyst. The surgical field was then thoroughly irrigated with approximately 2000 mL of hypertonic saline, maintaining a 15-minute dwell time to ensure complete scolicidal efficacy.

Histopathological examination confirmed the diagnosis of a hydatid cyst.​​ The patient was successfully discharged on postoperative day 6 without complications. Given the patient's pastoral lifestyle with frequent livestock (cattle/sheep) exposure and intraoperative hydatid cyst rupture, postoperative oral albendazole therapy (400 mg twice daily) was initiated at week 4 and continued for 24 weeks to prevent recurrence and potential disseminated implantation. Given these considerations, the patient underwent serial postoperative surveillance with abdominal ultrasound and hydatid antibody testing at one and six months, followed by CT scans and antibody testing at 12 and 24 months. Throughout this monitoring period, the patient exhibited sustained remission with no evidence of recurrence.

## Discussion

Hydatid cyst, caused by *Echinococcus* species (predominantly *E. granulosus*), is a parasitic infection. Transmission occurs through the accidental ingestion of eggs shed in the feces of definitive hosts (typically canids), which contaminate food, water, or soil. Following ingestion and hatching in the intestine, the released oncospheres penetrate the intestinal mucosa, enter the bloodstream or lymphatic system, and lodge in various organs to develop into hydatid cysts [4]. Typically manifesting as cystic lesions in the liver and lungs. However, in rare instances, it may involve other tissues, including skeletal muscle, accounting for merely 2-3% of all cases [2]. This rarity stems from inherent muscular resistance to parasitic colonization, attributable to elevated lactic acid concentrations and biomechanical clearance mechanisms from rhythmic contractions, both factors potentially inhibiting hydatid cyst development [5]. Consequently, these parasitic cysts predominantly localize to the trunk, neck, and lower extremities, where muscles exhibit reduced contractile activity and greater vascularization.

Clinically, the initial phase of muscular echinococcosis is typically asymptomatic [6]. The clinical manifestations and signs of hydatid cyst disease depend on the affected organ, anatomical location, and the impact on adjacent tissues [2]. In our case, the iliopsoas hydatid cyst exhibited prolonged subclinical progression within the deep anatomical compartment (a region with sparse neural innervation) shielded by abdominal and pelvic viscera, osseous structures, and fascial layers. Unlike hydatid cysts growing in superficial, palpable locations, its insidious growth pattern and non-specific clinical manifestations (such as chronic mechanical low back pain and vague abdominal discomfort) lead to delayed diagnosis, manifesting clinical features akin to a 'silent killer'. This condition often remains undetected until complications arise, such as cyst rupture, secondary bacterial infection, severe neural compression, or significant muscle destruction.

Compounding these diagnostic challenges are the limitations of current modalities. While serological testing serves as a valuable screening tool, it carries a non-negligible false-negative rate of 10-15%, further obscuring the diagnosis [7]. Typically, serology demonstrates high sensitivity (80-100%) and specificity (88-96%) for hepatic involvement, but its diagnostic utility declines for extrahepatic hydatid cysts (40-60%) [8]. This discrepancy may stem from the tripartite interplay of antigen exposure mechanisms, immune response characteristics, and limitations of detection methods. Isolated musculoskeletal hydatid disease frequently yields false-negative results, as these lesions are often primary without visceral involvement [9]. In our case, while the patient's serology was positive, even negative serology in endemic regions should heighten clinical suspicion.

Imaging plays a pivotal role in the diagnosis and staging of hydatid cyst. Ultrasonography is generally regarded as the first-line imaging modality for the diagnosis, staging, and follow-up of hydatid cysts, typically revealing unilocular or multilocular fluid-filled cysts with septations. However, CT and MRI serve as viable alternatives [10]. CT excels in detecting cyst calcifications and musculoskeletal involvement, usually demonstrating cystic lesions without significant wall or content enhancement post-contrast. Specific stages (e.g., CE4, CE5) may exhibit curvilinear or ring-like calcifications in the pericyst [11]. Conversely, MRI provides superior structural characterization: on T1-weighted imaging (T1WI), cyst fluid appears hypointense with isointense walls, while on T2-weighted imaging (T2WI), the fluid shows homogeneous hyperintensity with daughter cysts appearing even brighter, allowing clear visualization of intracystic architecture [12]. Both modalities enable precise anatomical delineation and spatial relationship assessment with adjacent tissues.

For muscular hydatid cyst cases, the most common treatment approach involves pharmacotherapy and surgical cyst excision. In non-operable cases, pharmacological therapy and/or PAIR (puncture, aspiration, injection, and reaspiration) serve as alternative therapeutic options [13]. The selection of each treatment modality is based on the cyst's structural characteristics, developmental stage, anatomical location, as well as the patient’s symptomatic presentation and overall clinical status. Surgical intervention is generally indicated for cysts exerting mass effect on adjacent organs, with hydatid cystectomy being the preferred procedure [14]. In our case, the patient's hydatid cyst was classified as CE2 according to the WHO diagnostic guidelines for echinococcosis [15]. The standard treatment protocol consists of preoperative albendazole therapy for 3-6 months followed by surgical resection (open or laparoscopic approach). However, due to pharmacodynamic limitations in deep tissue penetration, we opted for a shortened albendazole course. Early surgical intervention was prioritized to mitigate the risks of disease progression.

Intraoperative rupture of hydatid cysts is a severe complication that can lead to anaphylactic shock and widespread intraperitoneal dissemination. Therefore, preventing rupture is the paramount principle. However, if rupture occurs, immediate management must include: (1) anti-anaphylactic measures, (2) complete aspiration of spilled cyst fluid and daughter cysts, (3) thorough scolicidal irrigation, and (4) prevention of peritoneal implantation [16]. In our case, we implemented prompt anti-anaphylaxis protocols, used low-pressure suction to evacuate the spilled contents, and packed the ruptured area and surrounding tissues with hypertonic saline-soaked gauze. All contaminated instruments and drapes were replaced, followed by copious irrigation of the surgical field with hypertonic saline (maintained for 15 minutes of contact time). To prevent recurrent implantation, the patient received long-term postoperative albendazole therapy.

Antiparasitic therapy (albendazole/mebendazole) demonstrates efficacy against hydatid cysts [17]. Monotherapy can achieve a cure in 30-40% of cases and remains essential for inoperable disease, with its therapeutic value increasingly validated by evidence-based research over the past decade. Preoperative albendazole administration is critical for cyst sterilization, intracystic pressure reduction, and facilitation of subsequent interventions. For surgical cases, postoperative continuation of albendazole further decreases recurrence rates [18]. However, in our patient, the hydatid cyst was situated in poorly vascularized muscular tissue, where limited drug diffusion may have resulted in subtherapeutic concentrations. This prompted our decision to abbreviate the preoperative medical therapy, a factor that might have contributed to intraoperative cyst rupture. Postoperatively, the patient received standardized pharmacotherapy with favorable follow-up outcomes. This case highlights that managing the complexities of this disease requires not only guideline adherence but also indispensable individualized therapeutic considerations.

## Conclusions

Serological testing and advanced imaging modalities remain the cornerstone of preoperative diagnosis. While negative echinococcus-specific IgG does not exclude the disease, combining epidemiological exposure history with characteristic imaging findings can significantly enhance diagnostic confidence. Radical surgical resection serves as the therapeutic mainstay, though vigilance against intraoperative cyst rupture is imperative. Adjunctive anthelmintic therapy, both preoperatively and postoperatively, constitutes an essential component for preventing recurrence and mitigating procedural complications. This case underscores the critical need for heightened clinical suspicion in endemic regions and highlights the importance of a multimodal diagnostic approach, integrating clinical, radiological, and histopathological assessments to ensure timely diagnosis and management of this rare but clinically significant parasitic infection.
